# Risk of myocardial infarction following capecitabine treatment in patients with gastrointestinal cancer – a nationwide registry-based study

**DOI:** 10.1186/s40959-025-00401-x

**Published:** 2025-11-10

**Authors:** Jan Walter Dhillon Shanmuganathan, Kristian Kragholm, Harman Yonis, Morten Schou, Christian Torp-Pedersen, Laurids Østergaard Poulsen, Manan Pareek, Gunnar Gislason, Lars Køber, Dorte Nielsen, Tarec Christoffer El-Galaly, Peter Søgaard, Mamas A. Mamas, Phillip Freeman

**Affiliations:** 1 Department of Cardiology, Aalborg University Hospital, Hobrovej 18-22, Aalborg, 9000 Denmark; 2 Department of Clinical Medicine, Aalborg University, Aalborg, Denmark; 3 Herlev and Gentofte University Hospital, Copenhagen, Denmark; 4 Department of Clinical and Translational Research, Steno Diabetes Center Copenhagen, Herlev, Denmark; 5 Department of Oncology, Aalborg University Hospital, Aalborg, Denmark; 6 Department of Biomedical Sciences, Center for Translational Cardiology and Pragmatic Randomized Trials, Faculty of Health and Medical Sciences, University of Copenhagen, Copenhagen, Denmark; 7 Department of Cardiology, Copenhagen University Hospital Rigshospitalet, Copenhagen, Denmark; 8 Department of Cardiology, Copenhagen University Hospital Gentofte, Copenhagen, Denmark; 9 The Heart Centre, Copenhagen University Hospital - Rigshospitalet, Copenhagen, Denmark; 10 Department of Hematology, Aarhus University Hospital, Aarhus, Denmark; 11 Department of Clinical Epidemiology, Aarhus University Hospital, Aarhus, Denmark; 12 Department of Molecular Medicine, Aarhus University Hospital, Aarhus, Denmark; 13 Department of Clinical Medicine, Aarhus University, Aarhus, Denmark; 14 Department of Cardiology, Keele University, Keele, UK

**Keywords:** Capecitabine, Gastrointestinal cancer, Risk, Myocardial infarction, Mortality

## Abstract

**Background:**

Myocardial infarction (MI) is an adverse event potentially associated with capecitabine treatment, potentially impacting the continuation of cancer therapy. However, data on its incidence remain limited.

**Methods:**

Using Danish nationwide registries, we identified patients with gastrointestinal (GI) cancer treated with capecitabine (2004–2016) and compared them to age- and sex-matched controls without cancer. Patients with prior ischemic heart disease were excluded but were subsequently analyzed as a separate secondary cohort. Cumulative incidences of MI and competing risks were calculated, and multivariable regression analyses were performed.

**Results:**

A total of 23,820 patients with GI cancer treated with capecitabine and 47,640 controls without cancer were included. Differences in comorbidities and antianginal medications were not significantly different (*P* > 0.05 for all). At 6 months, the cumulative incidence of MI was significantly higher for patients treated with capecitabine at 0.6% (95% CI: 0.5%-0.7%) versus 0.3% (95% CI: 0.3%-0.4%) in controls, with a competing risk of death of 15.2% versus 0.7%. At 1 year, the cumulative incidence of MI for patients receiving capecitabine was 0.7% (95% CI: 0.6%-0.9%) versus 0.6% (95% CI: 0.5%-0.6%) among controls, with a competing risk for death of 29.7% versus 1.6%. After accounting for competing risks, the sub distribution hazard ratios indicated an increased risk of MI for patients receiving capecitabine compared with control subjects at both 6 months (hazard ratio: 2.02; 95% CI: 1.57–2.60; *P* < 0.001) and 12 months (hazard ratio: 1.28; 95% CI: 1.05–1.57; *P* = 0.015).

**Conclusion:**

Capecitabine treatment in GI cancer patients was associated with an increased MI risk at 6 and 12 months compared to controls, though absolute risks were low. Given the high competing mortality, the clinical relevance of this increased MI risk appears limited. In a secondary analysis of patients with known ischemic heart disease, our findings suggest that cardiovascular risk assessment and monitoring is warranted during capecitabine therapy, rather than routine avoidance of treatment.

**Supplementary Information:**

The online version contains supplementary material available at 10.1186/s40959-025-00401-x.

## Introduction

Fluoropyrimidines such as capecitabine, an oral prodrug of 5-fluorouracil (5-FU), are cornerstone chemotherapeutic agents in the treatment of gastrointestinal (GI) cancers [1–8], as well as metastatic breast cancer and head and neck cancers [9–11]. Cardiotoxicity is a recognized adverse effect, with the most common manifestations including chest pain, coronary artery spasm, and acute coronary syndrome, including myocardial infarction (MI). Less frequent but severe complications include cardiac arrhythmias, heart failure, cardiogenic shock, and sudden death [12–14]. The reported incidence of fluoropyrimidine-induced cardiotoxicity varies widely, ranging from 0 to 20% for 5-FU and 3% to 35% for capecitabine [15]. Serious cardiac events, including MI, cardiogenic shock, and cardiac arrest, occur in 0% to 2% of patients. Patients diagnosed with fluoropyrimidine-related chest pain, angina, or even MI often lack epicardial coronary obstruction on angiography [16]. The most prominent theory of fluoropyrimidine-related ischemia is therefore coronary vasospasm due to either endothelial dysfunction with defective formation of nitric oxide or endothelium-independent primary dysfunction of muscle cells [13, 17]. As capecitabine’s toxicity profile closely mirrors that of its active metabolite, 5-FU, it is presumed to share similar mechanisms of cardiotoxicity. In the literature, only few cases of MI resulting from capecitabine treatment have been reported, and there are no studies that prove a direct relationship between capecitabine and the acceleration of atherosclerosis [18–21]. However, this prior literature is limited by selected patient populations of small size and on case-reports. To address this gap, we conducted a nationwide registry-based study in Denmark to assess the 6- and 12-month risks of MI and mortality in patients with GI cancer treated with capecitabine compared with age- and sex-matched population controls.

## Methods

### Study population

We identified patients with gastrointestinal (GI) cancer who received capecitabine treatment between 2004 and 2016 using the Danish National Patient Registry. Of the 27,023 patients identified, 2,908 with pre-existing ischemic heart disease (IHD) were excluded, leaving 24,115 eligible patients. Among these, 295 could not be matched to controls, resulting in a final cohort of 23,820 patients, representing the largest cohort published thus far to assess cardiotoxicity of capecitabine. The same exclusion criterion was applied to matched population controls, and individuals with a prior diagnosis of ischemic heart disease were excluded from the control group before matching. A flowchart of the study population is provided in Supplemental Fig. 3.

Risk set matching was used to identify background population control subjects matched on age and sex and the year and month equivalent to capecitabine treatment initiation. This matching was conducted using risk set matching in a 1:2 ratio, with two controls selected for each patient receiving capecitabine, yielding 47,640 controls. Entry time in time-to-event analyses for patients and matched individuals from the background population was the date of first capecitabine administration for patients with GI cancer.

### Setting and data sources

We utilized a unique identifier, the Danish Civil Personal Registration number, which is allocated to every Danish citizen upon birth or immigration [22]. This unique identifier enables linkage on a personal level among nationwide registry data sources. The Danish Civil Registration System also includes data on date of birth and sex. Relevant data regarding selected in- and outpatient diagnosis codes were extracted from the Danish National Patient Registry to establish the presence of comorbidities including hypertension, hypercholesterolemia, diabetes, chronic obstructive pulmonary disease, heart failure, atrial fibrillation, and chronic kidney disease. Moreover, the Anatomical Therapeutic Chemical codes for selected prescription medication from the Danish National Prescription Registry were used to define certain comorbid conditions not solely on hospital contact diagnoses but also on whether certain prescription drugs were dispensed. Specifically, we defined diabetes and hypertension as either a relevant hospital-based diagnosis or dispensed prescription for antidiabetic or antihypertensive drugs, respectively.

For diabetes, we demanded at least one antidiabetic drug to be dispensed within a 180-day timeframe prior to study inclusion. Hypertension was defined when at least two prescribed antihypertensive drugs were dispensed in two consecutive 180-day periods before inclusion, as previously reported [22]. Additionally, data on specific antianginal medications such as nitrates, beta-blockers, and calcium blockers were also collected within a 180-day period prior to study inclusion, to examine potential differences in prescription drug use between patients on capecitabine and control subjects. Finally, the Danish Cause of Death Registry was utilized to gather information on the date and presumed cause of death. Details on the codes and definitions can be found in the Supplemental Tables 2,3 and 4.

### Outcomes

The primary outcome was myocardial infarction (MI) at 6 months and 1 year, defined strictly as acute myocardial infarction (ICD-10 codes I21.x), including ST-elevation MI (STEMI) and non-ST elevation MI (NSTEMI). Unstable angina was not included. The specific diagnostic codes used for MI are listed in Supplemental Table 2. Secondary outcomes were deaths from competing events other than MI, all-cause mortality and presumed cardiovascular cause of death. In a secondary analysis, we stratified capecitabine-treated patients based on the presence or absence of pre-existing ischemic heart disease (IHD) to compare MI risk between subgroups and against population controls. In accordance with numerous previous manuscripts, we defined cardiovascular death as any cardiovascular diagnosis registered as the cause of death in the Danish Cause of Death Register [23, 24].

### Statistics

Categorical variables were displayed utilizing frequencies and percentages, while continuous data were depicted using medians and interquartile ranges. The cumulative incidence of MI and overall mortality were evaluated utilizing the Aalen-Johansen and Kaplan–Meier estimates, respectively. To evaluate the absolute and relative risks of MI in patients treated with capecitabine compared with control subjects, a cause-specific Cox regression analysis was conducted. Based on Cox regression and average treatment effect modeling, outcomes were standardized for age, sex, comorbidity, and pharmacotherapy distributions of all included subjects. The specific covariates included age, sex, hypertension, hypercholesterolemia, diabetes, chronic obstructive pulmonary disease, chronic kidney disease, heart failure, atrial fibrillation, lipid-lowering drugs, aspirin, P12Y_12_ inhibitor treatment, vitamin K antagonist, direct oral anticoagulant therapy, and selected antianginal medications, including nitrates, beta-blockers, and calcium channel-blockers. Standardization ensured that the distributions of age, sex, comorbidities, and pharmacotherapies were equal between capecitabine-treated patients and control subjects. The Cox proportional hazards model adjusted for the aforementioned covariates was utilized to calculate the relative hazards. Additionally, a Fine-Gray regression analysis was performed to calculate the sub distribution hazard ratios, considering competing risks and adjusted for the same covariates. Additional analyses were made comparing capecitabine patients with and without pre-existing ischemic heart disease. Ischemic heart disease was defined accordingly to the ICD10 diagnosis where acute myocardial infarction, ST-segment elevation myocardial infarction and non-ST segment elevation myocardial infarction were included. The incident risks for MI within 5 days and 6 weeks after consecutive capecitabine administrations were also examined. Statistical significance was defined as a two-sided *P* value less than 0.05. Data managed was executed using SAS version 9.4 (SAS institute, Cary, NC, USA) and the analysis was conducted using R version 3.6.1 (R Core Team (2021). R: A language and environment for statistical computing. R Foundation for Statistical Computing, Vienna, Austria. URL https://www.R-project.org/).

### Ethics

The study was approved by the data responsible institute in the Capital Region of Denmark. In Denmark, ethical approval is not required for registry-based studies.

## Results

### Patients and characteristics

A total of 71,460 subjects were included in the final analysis, of whom 23,820 had GI cancer and were treated with capecitabine and 47,640 were age and sex matched controls.

There were no major differences in baseline characteristics between the two groups (Table 1). In addition, the percentage of patients treated with other chemotherapeutic agents, including irinotecan, oxaliplatin, bevacizumab, cetuximab, and panitumumab are also noted in Table 1. In supplemental analyses, patients with previous ischemic heart disease were included and characteristics of these patients were compared with patients without pre-existing ischemic heart disease as well as controls (Supplemental Table 1).

**Table 1:** Study population

	**Patients With GI Cancer**	**Control Subjects**	*p***-value**
	**(***n*** = 23,820)**	**(***n*** = 47,640)**	
Age	66 (59–73)	66 (59–73)	0.96
Male	11,130 (46.7)	22,260 (46.7)	1.00
Hypertension	8556 (35.9)	16,880 (35.4)	0.20
Hypercholesterolemia	4493 (18.9)	11,785 (24.7)	< 0.001
Diabetes	2389 (10.0)	4220 (8.9)	< 0.001
Chronic pulmonary obstructive disease	1264 (5.3)	2368 (5.0)	0.056
Heart failure	426 (1.8)	1399 (2.9)	< 0.001
Atrial fibrillation	1146 (4.8)	2384 (5.0)	0.27
Chronic kidney disease	232 (1.0)	733 (1.5)	< 0.001
Nitrates	485 (2.0)	1659 (3.5)	< 0.001
Calcium channel blockers	3144 (13.2)	6787 (14.2)	< 0.001
Beta-blockers	2590 (10.9)	6614 (13.9)	< 0.001
Lipid-lowering drugs	4244 (17.8)	11,173 (23.5)	< 0.001
Aspirin	2242 (9.4)	6985 (14.7)	< 0.001
P_2_Y_12_ Inhibitors	418 (1.8)	1245 (2.6)	< 0.001
Vitamin K antagonist	747 (3.1)	1490 (3.1)	0.97
Novel oral anticoagulations	300 (1.3)	591 (1.2)	0.86
Cotreated with irinotecan	678 (2.8)	NA	< 0.001
Cotreated with oxaliplatin	11,246 (47.2)	NA	< 0.001
Cotreated with bevacizumab	1995 (8.4)	NA	< 0.001
Cotreated with cetuximab	251 (1.1)	NA	< 0.001
Cotreated with panitumumab	21 (0.1)	NA	< 0.001

*GI* gastrointestinal, *NA* not available

Values are median (interquartile range) or *n* (%)

### Cumulative incidence of MI and Kaplan–Meier estimates of all-cause mortality

**Fig. 1:**
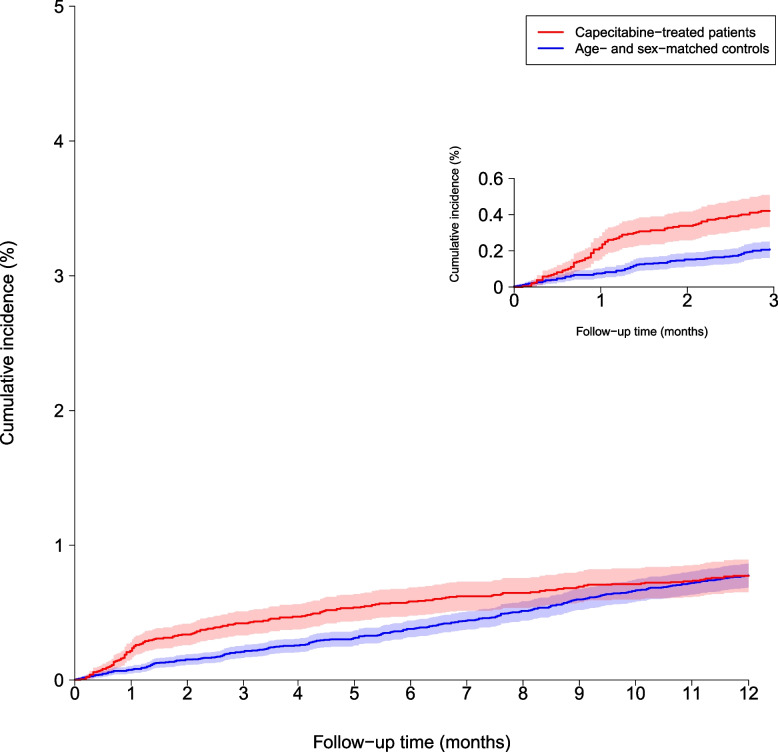
Cumulative incidence of myocardial infarction for capecitabine-treated patients versus age- and sex-matched population controls

**Fig. 2:**
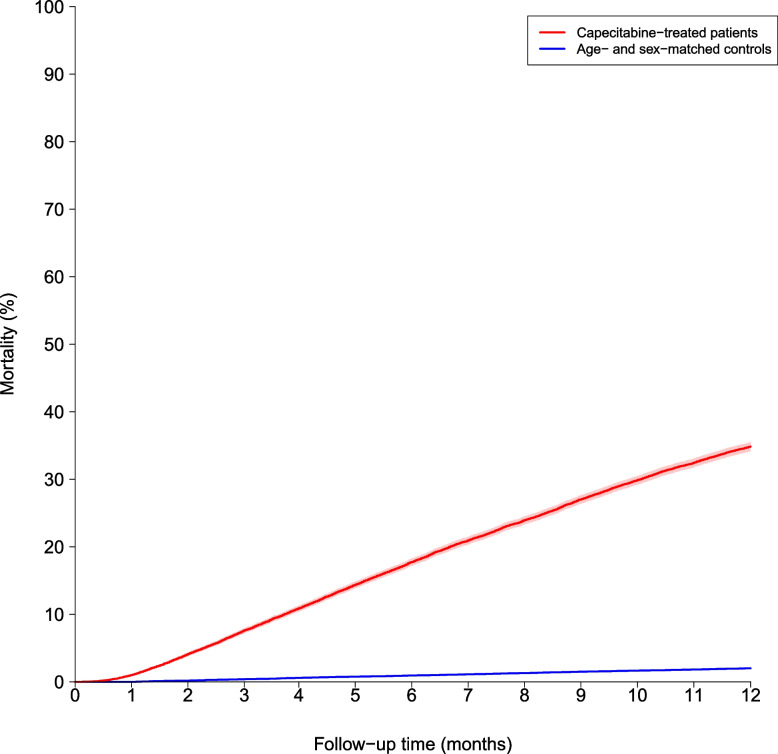
All-cause mortality for capecitabine-treated patients versus age- and sex-matched population controls

The loss to follow-up was low (0.1% for cases and 0.05% for controls) for the 1-year outcome. The 6-month cumulative incidence of MI was significantly higher for capecitabine-treated patients with GI cancer, at 0.5% compared with 0.3% among population control subjects (Fig. 1), with corresponding deaths from causes other than MI of 15.2% versus 0.7%, respectively. The 1-year cumulative incidences of MI were 0.7% and 0.6% for capecitabine treated patients and population control subjects, respectively (Fig. 1), with corresponding competing risks of death of 29.7% versus 1.6% (Fig. 2).

### Standardized and competing risk analysis

The standardized absolute 6 month risks of MI were 0.6% (95% CI: 0.5%−0.7%) for capecitabine treated patients versus 0.3% (95% CI: 0.3%−0.4%) for control subjects (*P* < 0.001). The corresponding standardized 1-year MI risks were 0.8% (95% CI: 0.7%−0.9%) versus 0.6% (95% CI: 0.5%−0.7%), respectively (*P* = 0.03). The average risk ratio standardized to the age, sex, selected comorbidity, antianginal, and lipid-lowering pharmacotherapy distributions of all subjects for the 6 months risk for MI for patients treated with capecitabine versus control subjects was 2.02 (95% CI: 1.53–2.48). The 1-year risk ratio for MI was 1.28 (95% CI: 1.03–1.52). To allow for censoring and competing risks, we also used Cox proportional hazards models to evaluate the risk for MI.

At 6 months, a Fine -Gray model to analyze adjusted sub distribution hazard ratios (HR) yielded nearly identical results. The HR was significant at 2.02 (95% CI: 1.57–2.60; *P* < 0.001), and similarly, at 12 months, the HR was statistically significant, but attenuated, at 1.28 (95% CI: 1.05–1.57; *P* = 0.015).

### Timing of MI

We also examined MI risk within the first three months following capecitabine initiation, as shown in Supplemental Fig. 1. This analysis revealed a higher cumulative incidence of MI in capecitabine-treated patients compared to matched controls within this early risk window.

The risks of MI within 5 days and 6 weeks following consecutive capecitabine administrations were evaluated. Within one year, 158 patients were diagnosed with MI, of whom 46 (29.1%) experienced MI within 5 days of capecitabine administration and 96 (60.8%) within 6 weeks. Furthermore, of the 46 patients diagnosed with MI within 5 days, 15 patients were rechallenged with capecitabine treatment without hospital admission for MI 3 months post MI, and all were alive 3 months after MI.

Supplemental Fig. 1 landmark analyzes where the cumulative incidence of myocardial infarction (MI) among capecitabine-treated patients compared to age- and sex-matched controls over the follow-up period from 0 to 3 months. Capecitabine treated patients exhibit a higher incidence of myocardial infarction within 3 months compared to control group patients. Supplemental Fig. 2 examines the cumulative incidence of myocardial infarction from 3 months to 1 year of follow-up among 90-day survivors who remained free of myocardial infarction during the first 90 days. Over time, a divergence emerges, with control patients displaying a higher cumulative incidence of MI compared to the capecitabine treated group which should been seen of the high mortality rate of capecitabine treated patients. Nonetheless, between 3 and 12 months of follow-up, a total of 71 MI (0.4%) events occurred in the capecitabine-treated group out of 18,988 patients, compared to 212 MI (0.6%) events in the control group out of 38,465 patients. During the same follow-up period, 5,478 deaths occurred in the capecitabine-treated group, compared to 575 deaths in the control group.

### MI risk in patients with pre-existing ischemic heart disease

**Fig. 3:**
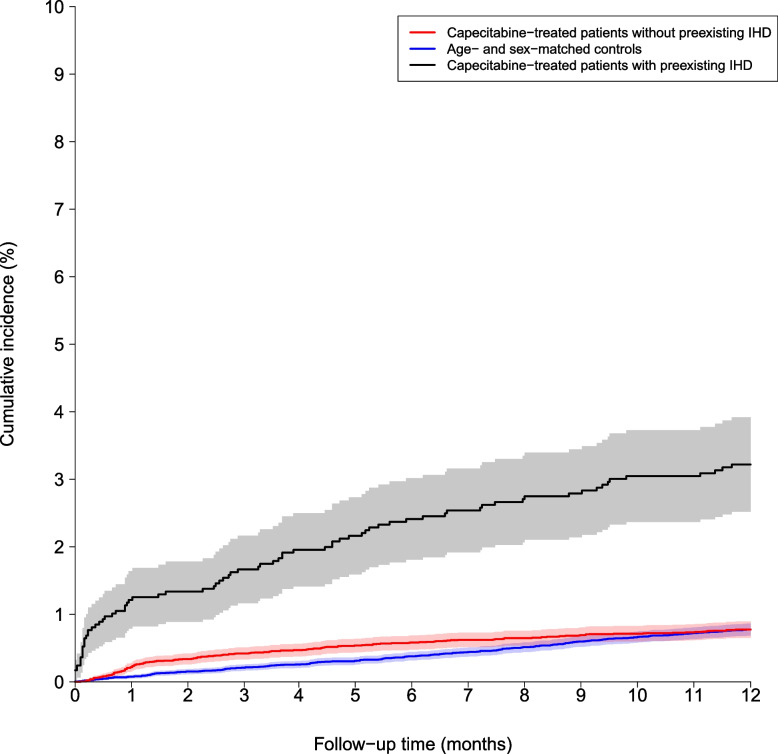
Cumulative incidence of myocardial infarction for capecitabine-treated patients with and without preexisting ischemic heart disease (IHD) versus age- and sex-matched population controls

To compare the relative and absolute MI risk across patient subgroups, we conducted a three-group analysis: capecitabine-treated patients without IHD, capecitabine-treated patients with IHD, and population controls. Figure 3 demonstrates that the cumulative incidence of MI in patients with pre-existing ischemic heart disease (n = 2,908) was more frequent compared with patients without pre-existing ischemic heart disease. The standardized absolute MI risks over a period of 180 days were 0.9% (95% CI: 0.6%−1.1%) versus 0.6% (95% CI: 0.5%−0.7% for patients with versus without pre-existing ischemic heart disease, while the corresponding standardized absolute MI risks over a period of 1 year were 1.1% (95% CI: 0.9%−1.5%) versus 0.8% (95% CI: 0.7%−0.9%). The corresponding standardized relative risk for MI among capecitabine-treated patients with pre-existing ischemic heart disease as compared with capecitabine-treated patients without pre-existing ischemic heart disease were 1.41 (95% CI: 0.92–1.91; *P* = 0.10) and 1.58 (95% CI:1.11–2.05; *P* = 0.02) for 6 months and 1 year, respectively.

### Cardiovascular death

The six month risk for presumed cumulative incidences for cardiovascular death for the control group was 0.3% (95% CI: 0.23%−0.32%), 1.7% (95% CI: 1.51%−1.83%) in capecitabine-treated patients without preexisting ischemic heart disease, and 3.4% (95% CI: 2.75%−4.09%) in capecitabine-treated patients with preexisting ischemic heart disease. At 1-year follow-up, the corresponding estimates were 0.6% (95% CI: 0.51%−0.65%), 2.7% (95% CI: 2.52%−2.93%) and 5.8% (95% CI: 4.99%−6.67%).

Further analyses showed that 1,723 patients have been exposed to both 5-FU and capecitabine. When excluding patients treated with both 5-FU and capecitabine, cardiovascular death at 6 months was 0.5% for patients treated with capecitabine without preexisting ischemic heart disease versus 0.3% for controls. For the 1-year risk of presumed cardiovascular death, the corresponding results were 0.7% versus 0.6%.

## Discussion

In this large, nationwide register-based study, we examined the incidence of MI and mortality in patients diagnosed with GI-cancer who underwent treatment involving capecitabine compared with age- and sex-matched controls. Although the 6- and 12-month risks for MI in capecitabine-treated patients was significantly higher than for controls, the absolute risk was limited, suggesting the clinical significance of these risks seem to be limited when considering the substantial competing risk of death in this population.

According to current studies, cancer is an independent risk factor for ACS, which is consistent with the increased MI incidence seen in patients on capecitabine. The occurrence of ACS is nearly twice as high in cancer patients compared to the general population with a cumulative incidence of MI of 2.0% versus 0.7 in controls [25, 26]. The highest incidence of ACS occurs within the first 6 months after a cancer diagnosis and is especially high in advanced-stage cancer patients which correlates with our finding of nearly 60.8% of MI within the first 6 weeks of capecitabine treatment. The mechanism of the cancer is that it induces a hypercoagulable state, increases systemic inflammation, and promotes endothelial dysfunction, all of which accelerate atherosclerosis and increase the likelihood of thrombotic events [27]. In our study MI risk was 1.58-fold higher in capecitabine treated patients with pre-existing ischemic heart disease which can indicate that cancer itself increases ACS risk which may be further amplified by capecitabine treatment.

The timing of MI events in relation to capecitabine treatment revealed critical insights. Among those diagnosed with MI, 29.1% had MI within 5 days of capecitabine treatment and 60.8% within 6 weeks. In line with previous reports highlighting the high risk of arterial thrombotic events during the first 90 days of cancer therapy, our 3-month landmark analysis confirmed that capecitabine-treated patients exhibited a substantially higher incidence of MI compared to controls (Supplemental Fig. 1). A similar timing was observed with 5-FU treatment, where 55.4% of MI events occurred within 5 days and 75.4% within 1 month of administration [28]. Our study shows the risk of MI shortly after administration of both chemotherapeutic agents. In support of our study findings, Kuppens et al. highlighted that cardiotoxicity is highly probable within 2–3 days after initiating capecitabine [29]. Furthermore, Polk et al. documented that cardiotoxicity first manifested in most patients during the initial cycles of treatment [15]. The early onset of cardiotoxicity is supported by previous research indicating that coronary artery vasospasm is the predominant mechanism because of either endothelial dysfunction with impaired nitric oxide production or endothelium-independent primary smooth muscle cell dysfunction underlying these events [13, 30].

Others have described accessory mechanisms that could lead to an acute imbalance between oxygen supply and demand. These mechanisms could include abnormal adenosine triphosphate consumption in cardiomyocytes or modifications in erythrocyte oxygen storage and transport.

The exact prevalence of ischemia linked to capecitabine is unclear however an incidence of 3% to 35% has been reported in published research as cardiotoxicity; this could be explained by variations in study sample sizes, broad definitions of cardiac events, patient-related risk factors such as age or cardiovascular risk factors, or treatment-related factors (such as dosage, mode of administration, or combination with other cardiotoxic drugs).

In our study, of the 46 patients diagnosed with MI within 5 days, 15 patients were rechallenged with capecitabine treatment without hospital admission for reinfarction 3 months post MI, and all were alive 3 months post MI. Similar findings were found in our study of intravenous 5-FU administration [28]. This seems feasible from a global perspective but still requires a personalized approach and shared decision-making between oncologist, cardiologist, and patient.

There are relatively few reported cases of acute coronary syndrome resulting from capecitabine treatment, and to date, large datasets evaluating the risk of MI over different time intervals are scarce. The duration of capecitabine treatment varies depending on the type and stage of cancer and the specific treatment plan, ranging from 6 months to several years [31, 32]. A systematic review by Polk et al. of 30 studies, including randomized controlled trials and observational studies, reported symptomatic cardiotoxicity in 3–35% of capecitabine-treated patients [15]. Common symptoms included chest pain, palpitations, dyspnea, and hypotension. Serious clinical events such as MI, cardiogenic shock, and cardiac arrest occurred in 0–2% of patients, consistent with our study findings. As such, even though patients treated with capecitabine have a higher risk of MI at 6 months and 1 year, when compared to the population control subjects, the absolute risk for MI is small and given the high mortality rate in this population, the clinical significance of these differences appears limited.

### Clinical implications and perspectives

While capecitabine treatment in patients with gastrointestinal cancer leads to a statistically significant relative risk increase of MI when compared to the general population, the study draws attention to that this risk remains very low in absolute terms, especially in consideration of the competing risk of death. In our study, we found that out of many patients who had MI, only a few—15 people—were safely given capecitabine again and, none of them experienced another MI while they stayed alive for at least 3 months after their initial MI.

Capecitabine treatment is not universally contraindicated for many patients with prior ischemic heart disease and some who experience a MI during therapy can still be treated. Risks may potentially be reduced by individualized risk assessments, and close monitoring, while necessary cancer treatments can be benefited from through the consideration of prophylactic antianginal therapy.

Drawing attention to the importance of early vigilance against cardiotoxicity, especially during the first weeks of treatment, the results reveal that almost one-third of MI events happen within just 5 days after capecitabine is given. Future studies should actively explore multiple mechanisms like vasospasm and assess multiple strategies for early detection and prevention to improve treatment protocols, balancing both effective cancer care and cardiovascular safety.

Furthermore, we observed a notable increase in cardiovascular mortality among capecitabine-treated patients compared with matched controls. This may reflect a combination of factors, including an underdiagnosis or misclassification of MI events, where sudden cardiac death or other cardiac-related symptoms may not have been captured as definite myocardial infarctions. In severely ill patients, especially those with advanced-stage cancer, the clinical focus may shift toward palliative care, and comprehensive cardiovascular evaluation may not be prioritized, leading to cardiovascular deaths potentially being underreported or misattributed [33]. Additionally, cancer patients are more vulnerable to arrhythmic events, heart failure, and stress-induced cardiomyopathy, which may be exacerbated by the cardiotoxic effects of capecitabine [34]. The increased cardiovascular mortality may also reflect the cumulative effect of systemic inflammation, a hypercoagulable state, and pre-existing cardiovascular comorbidities, all of which contribute to a heightened baseline risk of fatal cardiovascular events [35]. Thus, the observed cardiovascular mortality likely represents a broader spectrum of cardiotoxic effects beyond MI, emphasizing the need for enhanced cardiovascular surveillance and early intervention in this patient population.

### Limitations

The observational nature of our study has an inherent risk of bias including confounding. Information about treatment dose and cancer stage were not available. Choosing appropriate control subjects is a challenge in our study. Both tumor matched patients which is not treated with capecitabine and patients with other sub cancer types were considered inappropriate because of the burden of disease related competing risks. This statement is also acknowledge from an editorial of our previous paper [36].

The risk of MI may be enhanced by cancer due to the presence of inflammation and other common mechanisms and risk factors. Consequently, the risk of MI in patients with GI cancer who are being treated with capecitabine, when compared to a control group from the general population, may be influenced by confounding factors. Therefore, it is important to interpret our findings cautiously. However, differences in baseline and risk factor characteristics between capecitabine-treated patients and population controls were limited. We could not obtain the exact dose of capecitabine and therefore difficulties understanding whether there is dose-dependent relation between treatment and MI. Furthermore, data on cancer stage or treatment intent (e.g., adjuvant vs. palliative therapy) were not available in the Danish registries and thus could not be included in the analyses. This limits our ability to stratify the findings based on disease severity or prognosis. The relatively high all-cause mortality observed in the capecitabine-treated cohort may in part reflect a larger proportion of patients with advanced or metastatic disease. It is hard to clearly differentiate whether it is the cancer itself in the capecitabine treated patients or the capecitabine-induced treatment that may be the underlying mechanism for MI. A significant limitation of this study is that it was not possible to obtain blood test results such as troponins, making it challenging to determine whether the observed myocardial infarctions (MIs) in capecitabine-treated patients were due to true ischemia or secondary factors leading to troponin elevation. While some degree of selection bias may be present, whereby patients with known ischemic heart disease are screened out before initiation of capecitabine therapy, the substantial size of our subgroup with pre-existing IHD argues against significant exclusion of these patients in routine clinical practice.

Finally, due to the only marginally increased and overall low absolute risk, it is suggested that the administration of capecitabine is associated with only a minimal risk of MI.

## Conclusion

Among patients with GI cancer treated with capecitabine, the relative risk of MI was significantly higher at 6 and 12 months compared with matched controls from the general population. However, the absolute risk remained low, and its clinical significance appears limited in the context of the high competing mortality risk in this population. While capecitabine may contribute to an increased MI risk, the overall burden of cardiovascular events remains small, and its oncological benefits likely outweigh this potential risk for most patients. Reference to effectiveness of increased survival of capecitabine treatment in cancers treated.

## Supplementary Information

Supplementary Material 1.

## Data Availability

The data used in this study are stored securely at Statistics Denmark and are not publicly available due to Danish data protection regulations. Access to the data requires approval from the Danish Data Protection Agency and Statistics Denmark. Requests may be directed to the corresponding author.
